# SIGIRR Alleviates Intestinal Mucosal Damage in Severe Acute Pancreatitis via the TLR4 Signaling Pathway

**DOI:** 10.1016/j.jcmgh.2025.101608

**Published:** 2025-08-11

**Authors:** Yang Liu, Feng Zhou, Yanping Song, Shenglong Wei, Bowen Cheng, Dingwei Liu, Huifang Xiong, Yong Xie, Xiaojiang Zhou

**Affiliations:** 1 Department of Gastroenterology, Jiangxi Provincial Key Laboratory of Digestive Diseases, Jiangxi Clinical Research Center for Gastroenterology, Digestive Disease Hospital, The First Affiliated Hospital, Jiangxi Medical College, Nanchang University, Nanchang, Jiangxi Province, China; 2 Department of Quality Control, The First Affiliated Hospital of Nanchang University, Jiangxi Medical College, Nanchang University, Nanchang, Jiangxi Province, China

**Keywords:** Gut Microbiota, Intestinal Barrier, Severe Acute Pancreatitis, Single Immunoglobulin Interleukin-1-related Receptor, Toll-Like Receptor-4 Signaling Pathway

## Abstract

**Background & Aims:**

Intestinal barrier dysfunction plays an important role in the development of severe acute pancreatitis (SAP). The aim of our study was to investigate the role of single immunoglobulin IL-1 receptor-related molecule (SIGIRR) and Toll-like receptor-4 (TLR4) signaling pathways in SAP intestinal barrier dysfunction.

**Methods:**

Intestinal epithelial monolayer barrier model were established by using Caco2 and HIEC cells. The effects of lipopolysaccharide or ascites fluids from patients with SAP on intestinal epithelial barrier function were assessed. Intestinal epithelial cells and mouse models with SIGIRR overexpression and knockdown were constructed to explore the role of SIGIRR on the intestinal inflammation and the TLR4 signaling pathway.

**Results:**

SIGIRR expression was decreased in both intestinal epithelial cells and intestinal tissues during SAP. Overexpression of SIGIRR in intestinal epithelial cells reduced inflammation and enhanced intestinal barrier function, as evidenced by measurements such as electrical resistance and fluorescein permeability. In vivo, SIGIRR overexpression reduced intestinal mucosal injury in SAP and inhibited the TLR4 signaling pathway, whereas SIGIRR knockdown worsened these effects. Additionally, SIGIRR overexpression influenced gut microbiota composition, encouraging the growth of beneficial species and suppressing harmful pathogens.

**Conclusions:**

SIGIRR plays a protective role in SAP by preserving intestinal barrier integrity and negatively regulating the TLR4 signaling pathway. Targeting SIGIRR offers a novel approach for improving SAP.

## Graphical abstract

**Figure:**
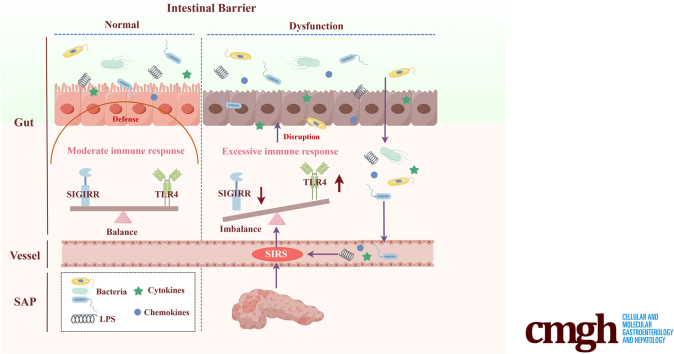

**Summary**

Single immunoglobulin IL-1 receptor-related molecule (SIGIRR) protects intestinal barrier integrity in severe acute pancreatitis by downregulating Toll-like receptor-4 signaling and reducing inflammation. Overexpression of SIGIRR enhances barrier function and modulates gut microbiota, highlighting its potential as a therapeutic target in severe acute pancreatitis.

Severe acute pancreatitis (SAP) is a common abdominal disease that carries a high risk of death, with a mortality of up to 30%.^1^^,^^2^ The disruption of intestinal barrier function in the early stage of SAP is the main reason for aggravation of the inflammatory response and leads to poor progression.^3^

The intestinal barrier is an important structure that prevents the invasion of pathogenic microorganisms. When the integrity of the intestinal barrier is compromised during SAP, the translocation of bacteria and lipopolysaccharide (LPS) leads to secondary infection of the pancreas and other organs. Subsequently, a large number of necrotic substances and inflammatory factors are produced to induce systemic inflammatory response syndrome (SIRS) and persistent multiple organ failure (MOF). Besselink et al confirmed that intestinal barrier dysfunction was significantly related to bacteremia and pancreatic necrosis in SAP and increased the mortality of patients.^4^ In contrast, maintaining intestinal mucosal homeostasis restored intestinal microbiota abundance and mitigated pancreatitis-associated infections.^5^, ^6^, ^7^

SAP intestinal barrier dysfunction involves the initiation of multiple injury factors, especially the release of multiple inflammatory mediators and the imbalance of cytokines. Studies have shown that the release of various cytokines is closely related to intestinal barrier dysfunction complicated by acute pancreatitis, and the Toll-like receptor-4 (TLR4) signaling pathway plays an important role in protecting the intestinal epithelial system. A series of studies have demonstrated that overactivation of the TLR4 signaling pathway occurs in multiple systems during SAP, which is closely associated with intestinal barrier dysfunction.^8^^,^^9^ Exogenous administration of lactic acid can reduce TLR4-mediated pancreatic inflammation.^10^ It has also been shown that the loss of intestinal epithelial TLR4 exacerbates pancreatic and intestinal damage.^11^

Single immunoglobulin IL-1 receptor-associated molecule (SIGIRR) is an immunomodulatory molecule belonging to the interleukin (IL)-1 receptor family. SIGIRR plays a negative regulatory role in the pathogenesis of inflammatory and immune diseases by interacting with IL-1 receptor type 1 (IL-1R1) and IL-1 receptor accessory protein (IL-1RAP) through heterodimerization of its extracellular domain.^12^^,^^13^ It was found that overexpression of SIGIRR could significantly reduce the secretion of inflammatory cytokines in LPS-stimulated lung epithelial cells.^14^ SIGIRR mutant mice developed a hypersensitive environment of intestinal TLR4, causing an early spontaneous intestinal inflammatory response.^15^ However, it remains unclear whether SIGIRR negatively regulates the TLR4 signaling pathway to reduce the intestinal barrier and subsequently improve SAP.

Therefore, this study aims to investigate the role of SIGIRR and TLR4 signaling pathways in SAP intestinal barrier dysfunction and explore the specific mechanism by which SIGIRR regulates SAP intestinal barrier to reduce pancreatic inflammation.

## Results

### Intestinal Mucosal Damage Was Observed in Mice With SAP

**Figure 1:**
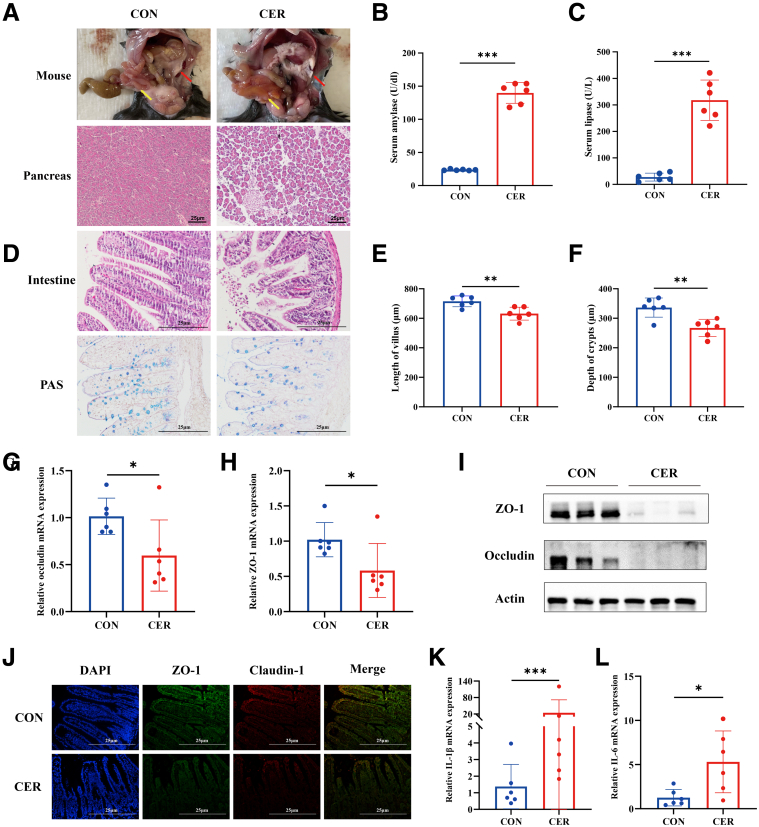
**Intestinal mucosal damage was observed in SAP mice.** (*A*) Gross observations of the pancreas (*red arrows*) and small intestine (*yellow arrows*), along with representative H&E-stained images. (*B–C*) Levels of serum amylase and lipase. (*D–F*) Representative H&E-stained and Periodic Acid-Schiff’s and Alcian Blue images of the small intestine, along with measurements of villus length and crypt depth. (*G–J*) Expression of intestinal barrier-related molecules, along with immunofluorescence staining images. (*K–L*) The mRNA levels of inflammatory factors in the small intestine. Data represent mean ± SEM from n = 6. ∗*P* < .05; ∗∗*P* < .01; and ∗∗∗*P* < .001.

The SAP model was established in mice through intraperitoneal injections of cerulein and LPS. The SAP group exhibited significant pancreatic edema, inflammatory cell infiltration, and necrosis (Figure 1*A*), alongside elevated serum amylase and lipase levels compared with controls (Figure 1*B–C*). The intestines also displayed lamina propria edema, mucus layer thinning, and villus shortening or loss (Figure 1*D–F*). Expression of intestinal barrier-related molecules, including Zonula occludens-1 (ZO-1) and Claudin-1, was downregulated, whereas inflammatory cytokines were significantly increased, indicating inflammatory damage to the intestinal mucosa in SAP mice (Figure 1*G–L*).

## LPS and Ascitic Fluid From Patients With SAP Induced the Disruption of Intestinal Barrier Function

The levels of inflammatory markers, enzymes, and endotoxins in biliary ascitic fluid (B-AF) and hypertriglyceridemic ascitic fluid (H-AF) from patients with SAP were measured using enzyme-linked immunosorbent assay (ELISA). Both B-AF and H-AF contained inflammatory factors, such as IL-1β, IL-6 and IL-8 (Table 1). Endotoxin levels in both fluids were below the detection limit of the assay kit. Amylase levels were significantly higher in B-AF than in H-AF, whereas adenosine dehydrogenase levels were lower.

**Table 1:** Detection of Components of AF From SAP

	IL-1β, pg/mL	IL-6, pg/mL	IL-8, pg/mL	IL-10, pg/mL	TNF-α, pg/mL	Endotoxin, EU/L	Amylase, U/L	Lactic dehydrogenase, U/L	Adenosine dehydrogenase, U/L
B-AF-1	37.9	>1000	120	202	10.7	<0.01	5842.1	1967.7	9
B-AF-2	22.4	>1000	48.5	33	17.5	<0.01	649.6	1057.5	8
H-AF-1	27.5	>1000	78	65.8	16.2	<0.01	77	1194.9	20
H-AF-2	54.3	>1000	3463	30.6	18.8	<0.01	148.1	945.2	12

AF, ascitic fluid; B-AF, biliary ascitic fluid; H-AF, hypertriglyceridemic ascitic fluid; IL, interleukin; SAP, severe acute pancreatitis; TNF, tumor necrosis factor.

**Figure 2:**
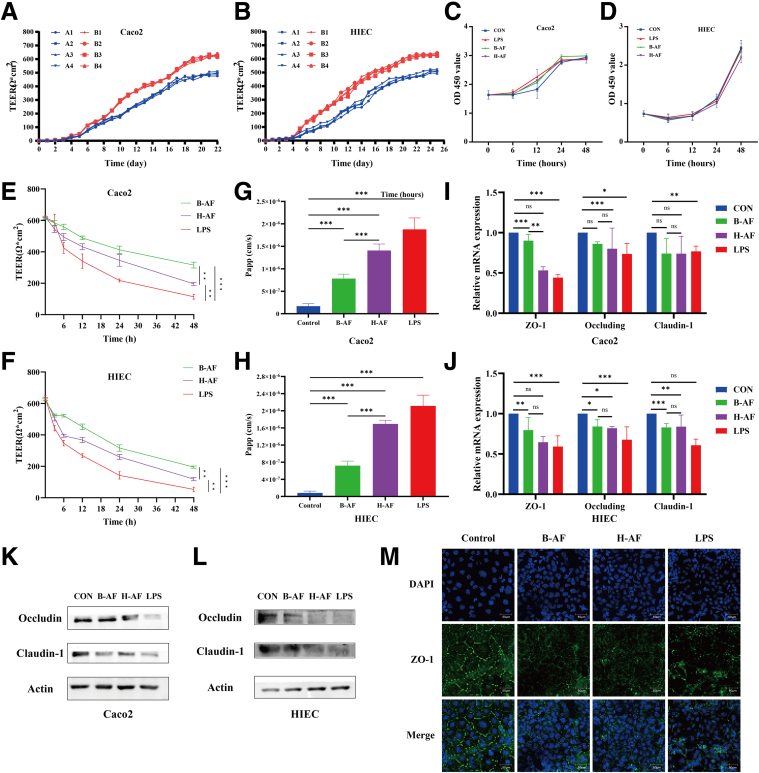
**LPS and AF from SAP patients induced the disruption of intestinal barrier function.** (*A–B*) Changes in the TEER of Caco2 monolayer cell barrier models with different seeding densities (A: 5000 cells/well; B: 10,000 cells/well) and HIEC models (A: 10,000 cells/well; B: 20,000 cells/well). (*C–D*) Changes in the viability of IECs after stimulation with LPS and AF were measured by the CCK-8 assay. (*E–H*) Changes in TEER and Papp in Caco2 and HIEC monolayer cell barrier models following stimulation by LPS or AF from patients with SAP. (*I–M*) Expression of intestinal barrier-related molecules, along with immunofluorescence staining images. ∗*P* < .05; ∗∗*P* < .01; and ∗∗∗*P* < .001.

Caco2 and human intestinal epithelial cells (HIECs) grew well after seeding, with transepithelial electrical resistance (TEER) gradually increasing and reaching a plateau on day 20 and day 22, respectively (Figure 2*A–B*). It is generally accepted that Caco2 monolayers are suitable for experiments when their TEER exceeds 260 Ω×cm^2^, and HIEC monolayers when their TEER exceeds 250 Ω×cm^2^.^16^^,^^17^ Both monolayer cell barrier models reached a TEER of 300 Ω×cm^2^ by day 10, meeting the criteria for successful model establishment.

LPS and ascitic fluid (AF) stimulation led to a significant time-dependent decline in TEER in Caco2 and HIEC cell barrier models (Figure 2*E–F*), with minimal impact on cell viability (Figure 2C–D). LPS, H-AF, and M-AF treatments caused a TEER drop of roughly 400 Ω×cm^2^ within 48 hours, compared with the milder effect of B-AF (*P* < .05). Fluorescence permeability in the model group significantly increased (Figure 2*G–H*), reflecting greater barrier permeability. We also observed that LPS and AF downregulated intestinal barrier-related molecule expression (Figure 2*I–M*). Interestingly, the damaging effect of B-AF on the intestinal epithelial barrier was significantly less than that of LPS and H-AF.

### LPS and AF From Patients With SAP Downregulated SIGIRR Expression

**Figure 3:**
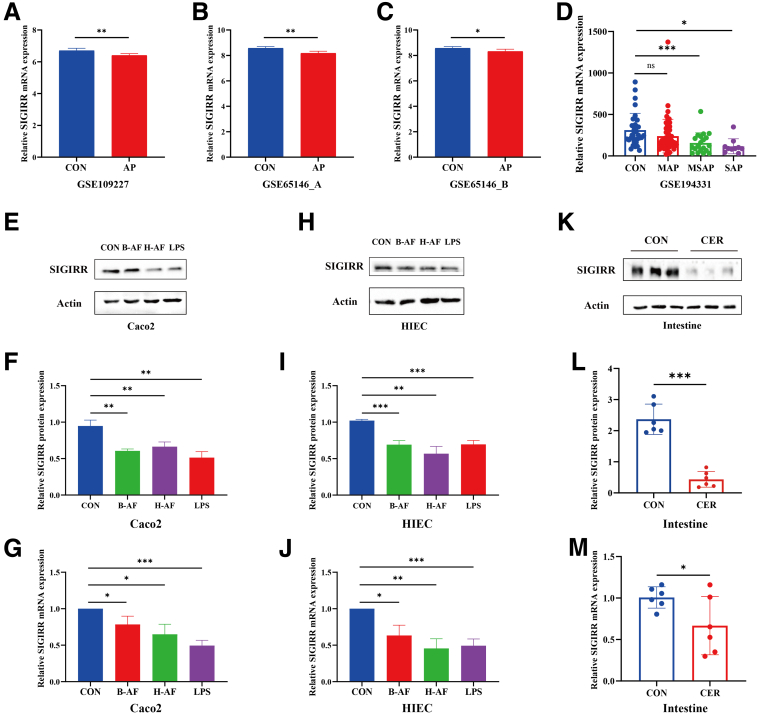
**LPS and AF from SAP patients downregulated SIGIRR expression.** (*A–D*) Expression of SIGIRR in datasets GSE109227, GSE65146, and GSE194331. (*E–M*) Expression of SIGIRR in IECs treated with LPS or AF from patients with SAP and in mouse intestinal tissues treated with cerulein. Data represent mean ± SEM from n = 6. ∗*P* < .05; ∗∗*P* < .01; and ∗∗∗*P* < .001.

Studies report that SIGIRR regulates inflammatory responses in the body. The Gene Expression Omnibus (GEO) datasets show that SIGIRR expression in the SAP group is significantly lower than in the control group during both the pancreatic injury and regeneration phases (Figure 3*A–C*). Additionally, SIGIRR expression in the peripheral blood of patients with pancreatitis is reduced and correlates positively with disease severity (Figure 3*D*).

Following stimulation with LPS or AF from SAP, the expression of SIGIRR mRNA and protein was downregulated in intestinal epithelial cells (IECs) (Figure 3*E–J*), as well as in the intestines of SAP mice (Figure 3*K–M*). LPS had the most pronounced effect on Caco2 cells, whereas H-AF showed a stronger effect on HIEC cells. Based on these findings, LPS and H-AF treatments were selected for further experiments.

### LPS and AF From Patients With SAP Activated the TLR4 Signaling Pathway

**Figure 4:**
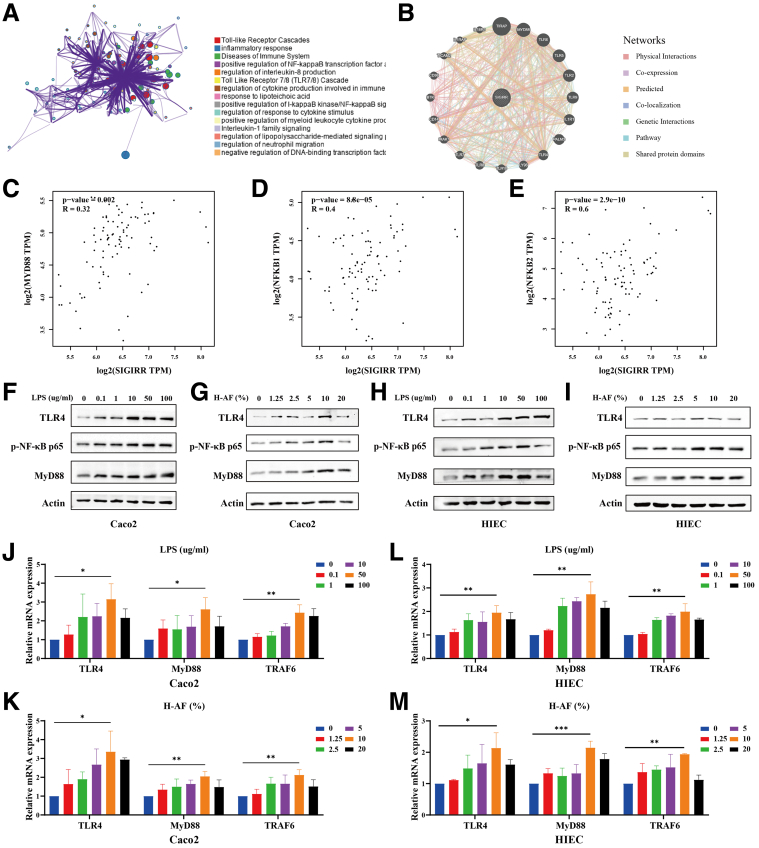
**LPS and AF from SAP patients activated the TLR4 signaling pathway.** (*A–E*) Enrichment analysis of SIGIRR using Metascape, gene interaction analysis using GeneMANIA, and correlation analysis using GEPIA. (*F–M*) Expression of the TLRs signaling pathway in IECs after treatment with varying concentrations of LPS and AF from patients with SAP. ∗*P* < .05; ∗∗*P* < .01; and ∗∗∗*P* < .001.

Enrichment analysis from Metascape and gene interaction analysis from GeneMANIA suggest that the TL4R signaling pathway may be involved in SIGIRR-regulated inflammatory responses (Figure 4*A–B*). Correlation analysis from Gene Expression Profiling Interactive Analysis (GEPIA) further supports a significant association between SIGIRR and key molecules in the TLR4 pathway, including TLR4, MyD88, and nuclear factor kappa-light-chain-enhancer of activated B cells (NF-κb) (Figure 4*C–E*).

We then investigated changes in the TLR4 signaling pathway during intestinal inflammation. LPS and AF treatments also activated the TLR4 signaling pathway. The expression levels of TLR4, MyD88, and NF-κB were significantly upregulated in the model group of IECs (Figure 4*F–M*).

### SIGIRR Alleviates Intestinal Mucosal Injury and Pancreatic Inflammation

**Figure 5:**
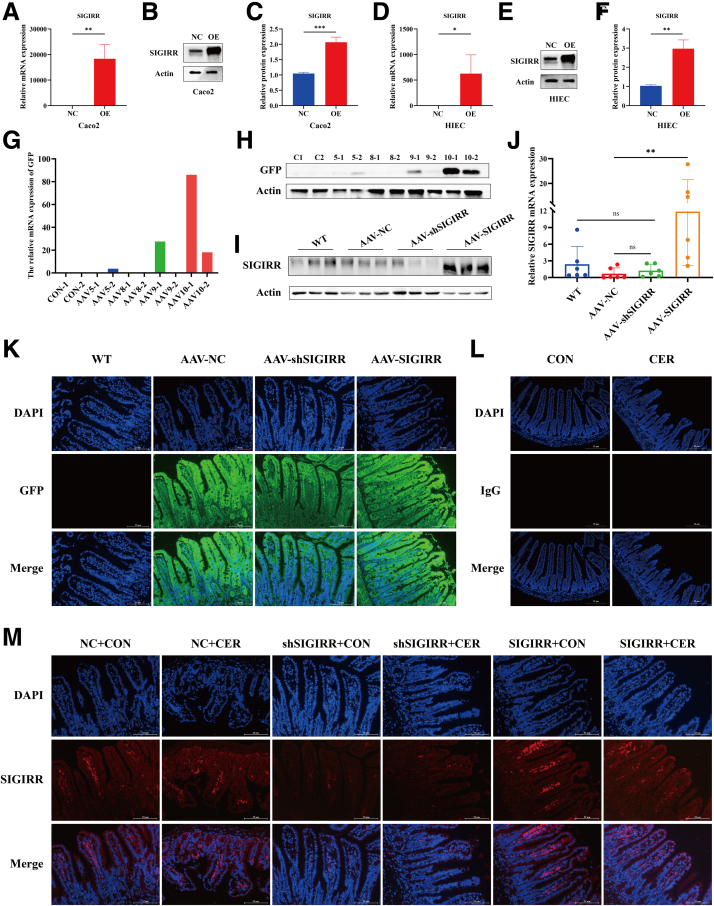
**Validation of SIGIRR expression in IECs with SIGIRR overexpression and mice with intestine-specific SIGIRR overexpression or knockdown.** (*A–F*) Expression of SIGIRR after lentiviral transduction of IECs overexpressing SIGIRR. (*G–H*) GFP expression in the intestinal tissues of mice after intraperitoneal injection with different serotypes of AAV. (*I–J*) Expression of SIGIRR in the distal ileum of mice following packaged AAV infection. (*K–M*) Immunofluorescence detection of intestinal GFP autofluorescence, nonspecific binding (IgG control), and SIGIRR expression. Data represent mean ± SEM from n = 6. ∗*P* < .05; ∗∗*P* < .01; and ∗∗∗*P* < .001.

The endogenous mRNA levels showed low SIGIRR expression, so only SIGIRR-overexpressing IECs were generated (Figure 5*A–F*). Four different adeno-associated virus (AAV) serotypes were administered via intraperitoneal injection to infect the intestinal tissues of mice over 4 weeks. AAV10 demonstrated the most effective targeting in the small intestine and was selected for vector construction (Figure 5*G–H*). The packaged AAV-shSIGIRR and AAV-SIGIRR were then reinjected intraperitoneally, followed by cerulein and LPS administration to induce the model (Figure 5*I–M*).

**Figure 6:**
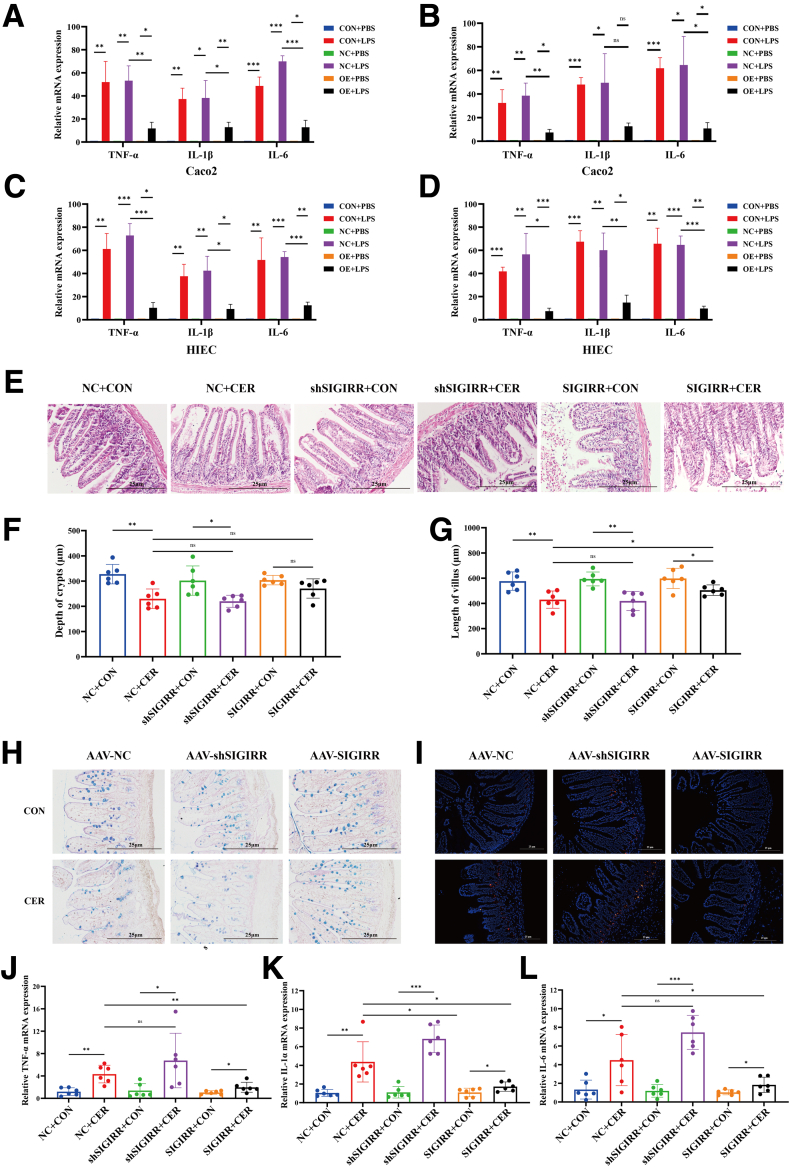
**SIGIRR alleviates intestinal inflammation during SAP.** (*A–D*) Expression of inflammatory cytokines in SIGIRR-overexpressing Caco2 cells stimulated by LPS or AF. (*E–G*) Representative H&E-stained images of the small intestine from SIGIRR-overexpression and knockdown mice, with measurements of villus length and crypt depth. (*H–I*) Detection by PAS-AB staining and EUB338 FISH. (*J–L*) Inflammatory cytokine expression in the small intestine of SIGIRR-overexpression and knockdown mice. Data represent mean ± SEM from n = 6. ∗*P* < .05; ∗∗*P* < .01; and ∗∗∗*P* < .001.

Overexpression of SIGIRR in IECs significantly reduced the inflammatory factor expression (*P* < .05) induced by LPS and H-AF (Figure 6*A–D*). SIGIRR also lead to a reduction in the expression of inflammatory factors tumor necrosis factor (TNF)-α, IL-1β, and IL-6 during SAP mice (Figure 6*J–L*).

**Figure 7:**
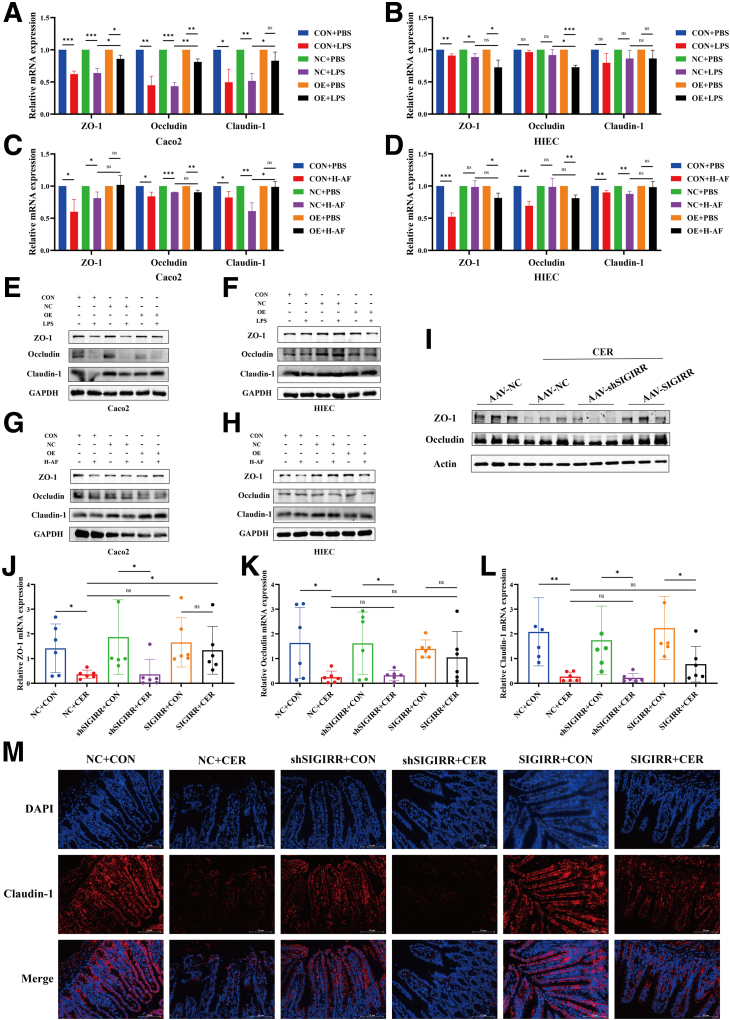
**SIGIRR plays a protective role in maintaining intestinal barrier.** (*A–H*) Expression of intestinal barrier-related molecules in IECs treated with LPS or AF from patients with SAP. (*I–M*) Expression of intestinal barrier-related molecules in the small intestine of SIGIRR-overexpression and knockdown mice. Data represent mean ± SEM from n = 6. ∗*P* < .05; ∗∗*P* < .01; and ∗∗∗*P* < .001.

AAV-SIGIRR infection reversed the shortening or loss of villi, lamina propria edema, and thinning of the mucus layer in the small intestine, which were exacerbated in the AAV-shSIGIRR SAP group (Figure 6*E–H*). The EUB338 universal bacterial probe revealed that SIGIRR knockdown compromised the intestinal barrier, allowing increased bacterial translocation across the mucosa into the lamina propria (Figure 6*I*). SIGIRR increased the expression of intestinal barrier-associated molecules ZO-1, Occludin, and Claudin-1 in LPS-treated IECs, particularly in Caco2 cells (Figure 7*A–H*). However, this effect was less pronounced in HIEC cells treated with H-AF.

In the AAV-SIGIRR group, a statistically significant increase in ZO-1 mRNA levels was observed in the intestines of mice, with nonsignificant increases in Occludin and Claudin-1 (Figure 7*J–L*). SIGIRR markedly elevated the protein levels of ZO-1 and Occludin, whereas shSIGIRR reduced them (Figure 7*I*). These results were also confirmed by immunofluorescence (Figure 7*M*).

**Figure 8:**
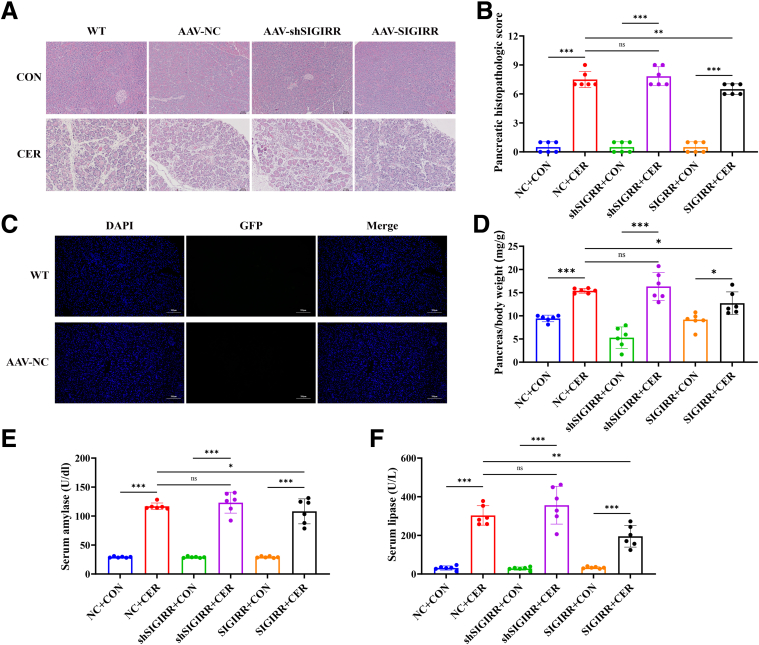
**SIGIRR improves the intestinal barrier and inhibits the TLR4 signaling pathway.** (*A–B*) Representative H&E-stained images (scale bar, 25 μm) of the pancreas, along with corresponding histopathological scoring. (*C*) Detection of pancreatic GFP autofluorescence. (*D*) Pancreatic/body weight ratio of mice. (*E–F*) Levels of serum amylase and lipase. Data represent mean ± SEM from n = 6. ∗*P* < .05; ∗∗*P* < .01; and ∗∗∗*P* < .001.

In addition to its effects on the intestine, SIGIRR also exhibited protective effects against pancreatic inflammation. Compared with the SAP group, the AAV-SIGIRR group showed a significant reduction in pancreatic pathology scores (Figure 8*A–B*). No green fluorescent protein (GFP) autofluorescence was detected in pancreatic tissues, demonstrating the intestinal-specific targeting of AAV (Figure 8*C*). Similar trends were observed in the pancreas-to-body weight ratio, as well as in serum amylase, lipase levels, and inflammatory markers (Figure 8*D–F*).

### SIGIRR Inhibited the Activation of the TLR4 Signaling Pathway in the Intestines During SAP

**Figure 9:**
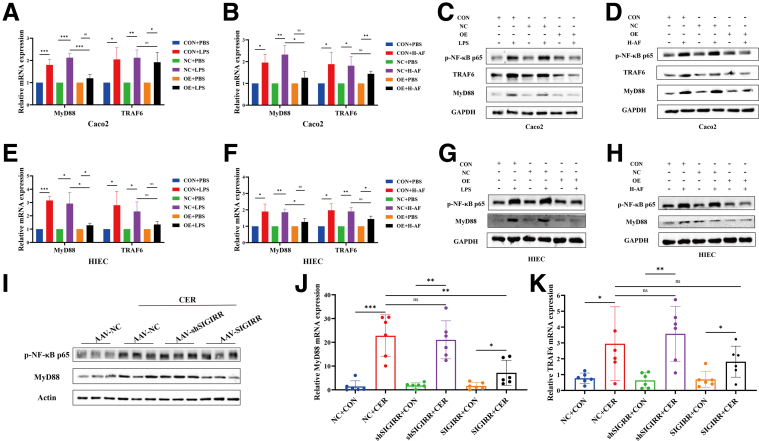
**SIGIRR inhibited the activation of the TLR4 signaling pathway in the intestines during SAP.** (*A–H*) Expression of TLR4 signaling pathway in SIGIRR-OE IECs treated with LPS or AF from patients with SAP. (*I–K*) Expression of TLR4 signaling pathway in the small intestine of SIGIRR-OE and knockdown mice. Data represent mean ± SEM from n = 6. ∗*P* < .05; ∗∗*P* < .01; and ∗∗∗*P* < .001.

We further investigated the effect of SIGIRR on the intestinal TLR4 signaling pathway. Overexpression (OE) of SIGIRR suppressed MyD88 mRNA expression in both IEC types (Figure 9*A–D*). In addition, the protein levels of p-NF-κB, MyD88, and TRAF6 were significantly reduced in the OE-SIGIRR group (Figure 9*E–H*).

Similarly, AAV-SIGIRR increased the expression of MyD88 mRNA but not TRAF6 (Figure 9*J–K*). SIGIRR also downregulated P-NF-κB and MyD88 protein levels in the small intestine of SAP mice, whereas AAV-shSIGIRR activated the TLR4 signaling pathway (Figure 9*I*). These findings suggest that the TLR4 signaling pathway may be involved in SIGIRR-mediated protection of the intestinal barrier and reduction of pancreatic inflammation.

### SIGIRR Modulates the Intestinal Microbiota in SAP Mice

**Figure 10:**
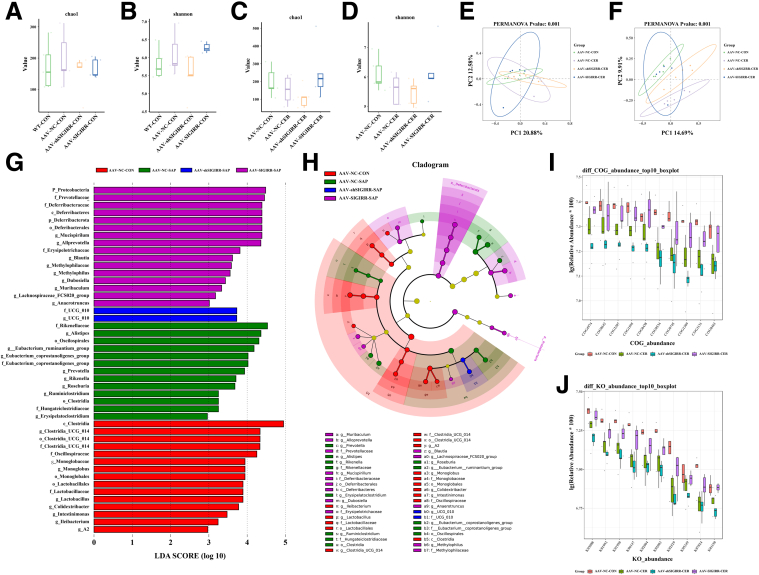
**SIGIRR modulates the intestinal microbiota in SAP mice.** (*A–F*) Analysis of α and β diversity in the gut microbiota of SIGIRR-OE and knockdown mice. (*G–H*) LEfSe analysis of the taxonomic unit information and rank tree of the intestinal microbiota in each group of mice. (*I–J*) Functional abundance analysis of COG and KO pathways in the gut microbiota across groups.

The intestinal microbiota of each group of mice was analyzed using 16S rRNA sequencing, and diversity was assessed at the ASV level. There were no statistically significant differences in the Chao1 and Shannon indices between groups, indicating that intraperitoneal injection of AAV does not affect the baseline intestinal microbiota in mice (Figure 10*A–B*).

In contrast, both indices significantly decreased in the SAP group, further declined in the AAV-shSIGIRR group, but increased in the AAV-SIGIRR group (Figure 10*C–D*). Beta diversity analysis also revealed differences in the gut microbiota composition among the 4 groups (Figure 10*E–F*).

LEfSe analysis revealed key differences in microbial populations among the groups (Figure 10*G–H*). Functional predictions based on COG and KO analyses of the gut microbiota indicated that SIGIRR can restore the functional abundance of the gut microbiota in SAP mice (Figure 10*I–J*), bringing it closer to the levels observed in the control group.

## Discussion

SAP is an inflammatory disease of the pancreas with high morbidity and mortality that is characterized by early activation of pancreatic enzymes in the acinar and complex cascade of inflammation.^18^ Sepsis secondary to pancreatic necrosis and peripancreatic infection is the most common cause of death.

It is generally believed that the injury of intestinal barrier leads to the transfer of intestinal pathogens and endotoxins from the intestinal lumen to distant organs in the early stage of SAP.^19^, ^20^, ^21^ Studies have shown a significant increase in both Gram-positive and Gram-negative bacteria, as well as anaerobic microorganisms, in the intestinal tissues of animals with acute necrotizing pancreatitis.^22^^,^^23^ The cell walls of Gram-negative bacteria contain LPS, which can stimulate an inflammatory response in epithelial cells. In the progression of SAP, a substantial accumulation of bacteria and their metabolites, particularly LPS, is critical to the intestinal damage observed in this condition. Our findings also confirmed that treatment with LPS significantly increases the permeability of the IEC barrier while reducing the expression of related barrier molecules.

SAP is associated with various extrapancreatic complications, including ascites, which can directly induce and aggravate inflammatory responses. Peritoneal drainage in SAP rat models significantly reduced intestinal mucosal damage and lowered serum endotoxin levels, suggesting that SAP ascites contributes to intestinal barrier dysfunction.^24^ To model the inflammatory environment in vitro, we used AF to stimulate an intestinal epithelial monolayer and observed decreased TEER and fluorescein permeability, along with significant downregulation of barrier-associated proteins, particularly in response to H-AF. Although we did not observe significant differences in inflammatory cytokine or endotoxin levels among different types of AF, the more pronounced barrier-disrupting effects of H-AF may be related to other components released during hypertriglyceridemic pancreatitis, such as proteases, reactive oxygen species, or arachidonic acid-derived metabolites.^25^, ^26^, ^27^ Patients with hypertriglyceridemia are more prone to persistent SIRS,^28^ and elevated triglyceride levels are independently linked to pancreatic necrosis,^29^ potentially leading to higher concentrations of damaging factors in ascitic fluid and greater intestinal barrier disruption compared with biliary pancreatitis.

SIGIRR not only inhibits tumor growth but also plays a role in immune and inflammatory diseases.^30^, ^31^, ^32^ Sampath et al^33^ found that in infants with necrotizing enterocolitis, genetic variations leading to SIGIRR loss-of-function have been shown to exacerbate LPS-induced inflammatory responses.^34^ The integrity of the intestinal mucosa is essential for maintaining proper gut barrier function. Inflammatory stimuli can disrupt tight junctions between IECs, leading to increased permeability and even epithelial cell necrosis.^35^ In addition, SIGIRR-deficient mice develop pronounced gastrointestinal inflammation and show increased susceptibility to a variety of enteric pathogens, such as the ulcerative colitis-associated pathobiont strain p19A.^36^ In our study, overexpression of SIGIRR in SAP mice significantly alleviated mucosal injury and reduced pancreatic inflammation, whereas knockdown SIGIRR abolished its protective effects against inflammation.

Our previous research found that ascites from SAP can upregulate TLR4 signaling pathways in macrophages, leading to NF-κB activation and subsequent release of inflammatory cytokines.^37^ Ascitic fluids contained a certain level of inflammatory cytokines, such as IL-1β and IL-8. In ascites-stimulated IECs and SAP mouse intestines, we also observed excessive activation of the TLR4 signaling pathway. SIGIRR overexpression reversed NF-κB activation, suggesting that SIGIRR may reduce intestinal mucosal injury and alleviate SAP by regulating TLR4 signaling pathway. This effect is likely related to the dual regulatory roles of SIGIRR: its extracellular domain can block the dimerization of IL-1R1 with IL-1R3/IL-1RAcP, whereas its intracellular TIR domain competitively binds to MyD88 dimers, thereby inhibiting downstream TLR signaling activation.^38^^,^^39^ The SIGIRR–MYD88 complex has also been found to activate IRAK1 kinase activity, thereby facilitating the expression of STAT3-dependent anti-inflammatory microRNAs.^40^

Moreover, the gut epithelium plays a crucial role in maintaining the symbiotic relationship between the host immune system and commensal microbes. Overexpression of SIGIRR increases the abundance of probiotics while reducing opportunistic pathogens. SIGIRR can affect gut microbiota composition by modulating the TLR signaling pathway, which influences the susceptibility of IECs. Studies have reported that mice deficient in MyD88 exhibit reduced production of antimicrobial peptides (AMPs) and mucus in IECs, rendering them highly susceptible to experimental colitis and enteric bacterial infections.^41^^,^^42^ Furthermore, conditional ablation of IKK subunits specifically in IECs leads to impaired NF-κB signaling, resulting in suppressed AMP expression and increased bacterial translocation across the colonic mucosa.^43^ Under inflammatory conditions, this microbial dysbiosis further exacerbates intestinal epithelial damage.

In conclusion, this study demonstrated that SIGIRR reduces mucosal injury, which in turn alleviates pancreatitis, with potential involvement of the TLR4 signaling pathway. These results offer new insights into the pathophysiology of SAP and the role of SIGIRR in regulating intestinal barrier function.

## Methods

### Cell Culture and Treatment

Human colon carcinoma Caco-2 cells (CL-0050, Pricella) and human normal intestinal mucosal epithelial HIEC cells (BNCC354805, BNCC) were used in the subsequent experiments. Caco-2 cells were cultured in Minimum Essential Medium (MEM; Gibco) supplemented with 1% nonessential amino acids (Solarbio) and 20% fetal bovine serum (FBS; Gibco). HIEC cells were cultured in Dulbecco’s Modified Eagle Medium (DMEM; Gibco, USA) supplemented with 10% FBS. LPS (L2880, Sigma) and ascitic fluid at different concentrations were added to stimulate IECs. Experiments were repeated 3 times with 3 triplicates.

### Animal Experiments

Male C57BL/6 mice (6- to 8-weeks old) were obtained from Gempharmatech Co Ltd and bred in-house under specific pathogen-free (SPF) conditions. All mice were maintained on a 12-hour light/12-hour dark cycle with free access to water and standard rodent chow. The mice were randomly divided into groups, with 6 mice in each group. Cerulein (100 μg/kg, 6264/1, TOCRIS) was administered intraperitoneally to each mouse once every hour for a total of 10 consecutive injections.^44^ The final injection was performed intravenously with LPS (5 mg/kg, ABS42020800, Absin). Mice were euthanized via CO_2_ inhalation 24 hours after the first intraperitoneal injection. Peripheral blood, pancreatic tissue, and intestinal tissue from the ileum near the ileocecal valve were collected. The study was approved by the Ethics Committee of the First Affiliated Hospital of Nanchang University ((2023) CDYFYYLK (02-031)).

### Collection of Ascitic Fluid

AFs were derived from 4 patients with SAP (Balthazar CT grade D or above and/or accompanied by other organ dysfunction) under aseptic conditions. Among them, 2 patients were diagnosed with hypertriglyceridemic pancreatitis, and their AFs were designated as H-AF. The remaining 2 patients had biliary pancreatitis, and their samples were referred to as B-AF. The collected ascitic fluid was immediately centrifuged at 3000 g for 15 minutes at 4°C. The supernatant was filtered 3 times through 0.45-μm and 0.22-μm membrane filters. Subsequently, 1 mL of the filtrate was spread onto bacterial culture agar plates and incubated at 37°C for 3 days. If no bacterial growth was observed under the microscope, the sample was deemed suitable for further use. Endotoxin levels were measured using the Kinetic Turbidimetric LAL Kit for Endotoxin Detection (KT0517, BIOENDO), and cytokine levels were assessed using a 12-plex cytokine assay kit (RAISECARE).

### Data Collection

High-throughput bulk sequencing datasets related to acute pancreatitis were screened in the GEO database. The GSE109227 dataset includes cerulein-induced mouse models. GSE65146 covers different stages of pancreatic regeneration following inflammatory injury, with 3 to 48 hours classified as stage A, and 60 hours to 144 days classified as stage B. The GSE194331 dataset includes peripheral blood gene expression data from patients with acute pancreatitis of varying severity.

### Construction and Transfection of a Lentivirus Vector

Lentivirus particles (Genechem) were produced by cotransfection of recombinat plasmids and lentivirus packaging auxiliary plasmids. Caco2 and HIEC cells were cocultured with lentivirus suspensions of different concentrations. After determining the appropriate transfection concentration, puromycin was used for drug screening to obtain stable SIGIRR overexpression Caco2 and HIEC cell lines.

### Measurement of Transepithelial Electrical Resistance

TEER was measured to assess the barrier integrity of intestinal epithelial monolayer barrier model. Caco2 cells and HIEC cells were seeded in Transwell chambers (Costar) with a surface area of 0.33 cm^2^ and pore size of 0.4 μm. The cells were cultured for 10 days in the medium and completely differentiated. The TEER values were recorded at 0, 3, 6, 12, 24, and 48 hours after coincubation with LPS and AF, which were added to the apical side of the epithelial cell monolayers, using a Millicell ERS apparatus (Millipore Co).

### Measurement of Paracellular Permeability

The permeability of the Caco2 and HIEC cell monolayers was measured by the flux of lucifer yellow (LY) (Beyotime, China). LY was added to the apical chamber in Hank’s balanced salt solution (HBSS). At the end of the incubation periods, the apical and basolateral solutions were collected, and the fluorescence signal was detected by a microplate reader at an excitation wavelength of 428 nm and an emission wavelength of 536 nm. Paracellular permeability (Papp), expressed as the apparent permeability coefficient, was calculated according to the following equation: Papp = (dQ/dT)/(ACo). dQ/dT represents the amount of LY molecules transported from the apical to basolateral chamber per unit of time (ug/s); A represents the surface area of the membrane (cm^2^); and Co represents the concentration of LY added.

### Construction and Transfection of AAV

The most suitable AAV serotype for targeting the small intestine was selected based on intraperitoneal injection of 5 AAV serotypes (Vigene Bioscience). AAV10-HSPA8 (NM_031165) and AAV10-SKP2 (NM_013787) overexpressing adeno-associated viruses were constructed. The intestinal tract of the mice was infected with AAV via intraperitoneal injection for 4 weeks, followed by treatment with cerulein.

### Measurements of Amylase and Lipase Activity

Blood was collected by cardiac puncture and centrifuged at 3000 rpm for 10 minutes to collect serum. Serum amylase was measured using commercially available kit (BC5055, Solarbio). Lipase activity was determined using specific detection kits (A054-2-1, Jiancheng Biotech).

### Reverse Transcription Polymerase Chain Reaction

RNA from Caco2 cells and HIEC-6 cells was extracted using an RNA extraction kit (19221ES, Yeason). Total RNA was subjected to reverse transcription using a Reverse Transcription kit (R333-01, Vazyme, China). Quantitative polymerase chain reaction (PCR) was performed utilizing the Hieff qPCR SYBR Green Master Mix (11201ES03, Yeason). The primer sequences used are shown in Table 2.

**Table 2:** Primers for Real-time PCR Analysis

Gene	Species	Primer	Sequence (5′-3′)	Species	Primer	Sequence (5′-3′)
β-actin	Human	Forward	AGAGAGGCATCCTCACCCTG	Mouse	Forward	GTGACGTTGACATCCGTAAAGA
		Reverse	GATAGCACAGCCTGGATAGCA		Reverse	GTAACAGTCCGCCTAGAAGCAC
SIGIRR	Human	Forward	CCCGAGGACCGCAAGTT	Mouse	Forward	TCCGTGACTCCTTCCTCTGATT
		Reverse	CCGAAAGCACCACGATGAG		Reverse	ACGATTAGCATGGGATCTTTGTC
TRAF6	Human	Forward	TTTGCTCTTATGGATTGTCCCC	Mouse	Forward	GCTTTGCGTCCGTGCGAT
		Reverse	CATTGATGCAGCACAGTTGTC		Reverse	GTCCGAATGGTCCGTTTGAG
MYD88	Human	Forward	GGCTGCTCTCAACATGCGA	Mouse	Forward	TGACCCCACTCGCAGTTTGT
		Reverse	CTGTGTCCGCACGTTCAAGA		Reverse	TTTGTTTGTGGGACACTGCTTTC
TLR4	Human	Forward	AGACCTGTCCCTGAACCCTAT	Mouse	Forward	TGAGGACTGGGTGAGAAATGAGC
		Reverse	CGATGGACTTCTAAACCAGCCA		Reverse	CTGCCATGTTTGAGCAATCTCAT
L-1β	Human	Forward	GCCACCTTTTGACAGTGATGAG	Mouse	Forward	AGGCTCCGAGATGAACAACAAA
		Reverse	AAGGTCCACGGGAAAGACAC		Reverse	GTGCCGTCTTTCATTACACAGGA
IL-6	Human	Forward	TAGTCCTTCCTACCCCAATTTCC	Mouse	Forward	CCCCAATTTCCAATGCTCTCC
		Reverse	TTGGTCCTTAGCCACTCCTTC		Reverse	CGCACTAGGTTTGCCGAGTA
TNF-α	Human	Forward	GACGTGGAACTGGCAGAAGAG	Mouse	Forward	ACCCTCACACTCACAAACCA
		Reverse	TTGGTGGTTTGTGAGTGTGAG		Reverse	ATAGCAAATCGGCTGACGGT
ZO-1	Human	Forward	CAGAGCCTTCTGATCATTCCA	Mouse	Forward	GCCGCTAAGAGCACAGCAA
		Reverse	CATCTCTACTCCGGAGACTGC		Reverse	TCCCCACTCTGAAAATGAGGA
Occludin	Human	Forward	AAGAGTTGACAGTCCCATGGCATAC	Mouse	Forward	TTGAAAGTCCACCTCCTTACAGA
		Reverse	ATCCACAGGCGAAGTTAATGGAAG		Reverse	CCGGATAAAAAGAGTACGCTGG
Claudin-1	Human	Forward	GCCAGGTACGAATTTGGTCAG	Mouse	Forward	ATGTGGATGGCTGTCATTGGG
		Reverse	TTGGTGTTGGGTAAGAGGTTGT		Reverse	GGACAGGAGCAGGAAAGTAGGA

PCR, polymerase chain reaction.

### Western Blot Analysis

Total proteins were extracted from Caco2 cells and IEC-6 cells with RIPA lysis buffer (R020, Solarbio). Protein concentrations were determined by a BCA protein assay kit (23225, Thermo Fisher). The proteins were separated using 10% SDS–PAGE and electrophoretically transferred to NC membranes. After blocking with 5% bovine serum albumin (BSA) for 1 hour, the membranes were incubated with primary antibodies overnight at 4°C. Primary antibodies are listed in Table 3. Subsequently, the membranes were incubated with an horseradish peroxidase (HRP)-conjugated secondary antibody for 1 hour at room temperature. The blots were visualized with an enhanced chemiluminescence kit (ECL, Thermo Fisher).

**Table 3:** Primary Antibodies

Antibodies	Source	Cat NO.
SIGIRR	Abcam	ab25875
TLR4	Santa Cruz Biotechnology	sc293072
MyD88	Abcam	ab135693
TRAF6	Abcam	ab13853
p-NF-κB p65	Cell Signaling Technology	3033S
ZO-1	Proteintech	21773-1-AP
Claudin-1	Proteintech	28674-1-AP
Occludin	Proteintech	27260-1-AP
GAPDH	Proteintech	60004-1-Ig
β-Actin	Transgen	HC201-01

### Histological Analysis

Pancreas tissue samples were fixed in 10% formalin for 24 hours, embedded in paraffin, and sectioned. The sections were processed for hematoxylin and eosin (H&E) staining. Pancreatic histopathology was independently scored based on Schmidt et al on a scale of 0 to 12 in 3 items by 2 pathologists.^45^

### Immunofluorescence Assay

Cells on the coverslips were fixed in 4% paraformaldehyde methanol for 30 minutes, washed 3 times with phosphate buffered saline (PBS) and blocked with 5% BSA. Small intestinal sections were fixed using paraformaldehyde and permeabilized with Triton X-100 in PBS, followed by subsequent blocking with blocking reagents. Samples were incubated with a specific primary antibody at 4°C overnight, washed, and then incubated with their respective secondary fluorescein-conjugated antibodies. Nuclei were stained with 4′,6-diamidino-2-phenylindole (DAPI; C1002, Beyotime). Images were captured using fluorescence microscope (Nikon).

### Fluorescence In Situ Hybridization

Bacterial translocation was detected by fluorescence in situ hybridization (FISH) (FB0016, Future Biotech, China). After dewaxing distal ileum tissues, hybridization was performed overnight at 52°C with Cy3-conjugated EUB338 probe (5'-GCTGCCTCCCGTAGGAGT-3'). Sections were then washed, counterstained with DAPI, and visualized under an Olympus fluorescence microscope.

### Sequencing to Determine Gut Microbiota Diversity

Fresh mouse feces from each group were snap-frozen and stored at −80°C. Bacterial DNA was extracted using the HiPure Stool DNA Kit B (Magen), and DNA concentration and integrity were assessed and agarose gel electrophoresis. The V3-V4 regions of the bacterial 16S rRNA gene were amplified using universal primers, and the purified PCR products were sequenced on an Illumina NovaSeq6000. Sequences were annotated and BLAST searched against the Silva database (Version 138) using the q2-feature-classifier. Sequencing of the 16S rRNA gene amplicon were performed by OE Biotech Co, Ltd.

### Statistical Analysis

The statistical analyses were performed using SPSS software (version 26.0). Continuous data are presented as mean ± standard deviation (SD). For normally distributed data with equal variance, we used Student’s *t*-test or 1-way analysis of variance (ANOVA) followed by Tukey’s post-hoc test. For non-normally distributed data or when variance assumptions were violated, appropriate nonparametric tests were applied. A *P*-value < .05 was considered statistically significant.
